# Adverse events at the end of life of hospital patients with or without a condition relevant for palliative care: a nationwide retrospective record review study in the Netherlands

**DOI:** 10.1186/s12904-024-01461-z

**Published:** 2024-06-10

**Authors:** B. Schouten, S. M. van Schoten, F. M. Bijnsdorp, H. Merten, P. W.B. Nanayakkara, A. K.L. Reyners, A. L. Francke, C. Wagner

**Affiliations:** 1 Department of Public and Occupational Health, Amsterdam UMC, Vrije Universiteit Amsterdam, Amsterdam Public Health Research Institute, P/O Box 7057, De Boelelaan 1117, Amsterdam, 1007 MB The Netherlands; 2 Netherlands Institute for Health Services Research (Nivel), Utrecht, The Netherlands; 3 Section General Internal Medicine, Department of Internal Medicine, Amsterdam UMC, Vrije Universiteit Amsterdam, Amsterdam Public Health Research Institute, De Boelelaan 1117, Amsterdam, The Netherlands; 4 Department of Medical Oncology, Center of Expertise in Palliative Care, University Medical Centre Groningen, University of Groningen, Groningen, The Netherlands; 5 Expertise Center Palliative Care, Amsterdam UMC, Amsterdam, The Netherlands

**Keywords:** Patient safety, Medical errors, Palliative care, End-of-life, Adverse events, Hospitals

## Abstract

**Background:**

Patient safety is crucial for quality of care. Preventable adverse events (AEs) occur in 1 of 20 patients in the hospital, but it is unknown whether this is different for patients with a condition relevant for palliative care. The majority of the limited available research on this topic is only focused on patients already receiving palliative care, and do not make comparisons with other patients at the end-of-life. We identified and compared the prevalence, preventability, nature and causes of AEs in patients with and without a condition relevant for palliative care.

**Methods:**

A nationwide retrospective record review study was performed in 20 Dutch hospitals. A total of 2,998 records of patients who died in hospital in 2019 was included. Records were reviewed for AEs. We identified two subgroups: patients with (*n* = 2,370) or without (*n* = 248) a condition relevant for palliative care through the selection method of Etkind (2017). Descriptive analyses were performed to calculate prevalence, nature, causes and prevention strategies. T-tests were performed to calculate differences between subgroups.

**Results:**

We found no significant differences between subgroups regarding AE prevalence, this was 15.3% in patients with a condition relevant for palliative care, versus 12.0% in patients without a condition relevant for palliative care (*p* = 0.148). Potentially preventable AE prevalence was 4.3% versus 4.4% (*p* = 0.975). Potentially preventable death prevalence in both groups was 3.2% (*p* = 0.938). There were differences in the nature of AEs: in patients with a condition relevant for palliative care this was mostly related to medication (33.1%), and in patients without a condition relevant for palliative care to surgery (50.8%). In both subgroups in the majority of AEs a patient related cause was identified. For the potentially preventable AEs in both subgroups the two most important prevention strategies as suggested by the medical reviewers were *reflection and evaluation* and *quality assurance.*

**Discussion:**

Patient safety risks appeared to be equally prevalent in both subgroups. The nature of AEs does differ between subgroups: medication- versus surgery-related, indicating that tailored safety measures are needed. Recommendations for practice are to focus on reflecting on AEs, complemented with case evaluations.

**Supplementary Information:**

The online version contains supplementary material available at 10.1186/s12904-024-01461-z.

## Background

Delivering high quality hospital care implies that care must be safe. Ensuring patient safety is a worldwide challenge, preventable adverse events (AEs) occur in 1 of 20 hospitalized patients [1]. In the Netherlands, the incidence of AEs in patients who died at hospitals in 2019 was 14.6%, and 4.2% for preventable AEs [2]. AEs can be defined as “an unintended injury that results in prolonged hospital stay, disability or death, and is caused by healthcare management rather than disease” [3]. Research on the nature and preventability of AEs can be used to reduce the incidence of patient harm and improve quality of care. In this study we focus on patient safety and AEs in a specific population: patients who died in hospital.

A literature review showed that for most people at the end of live their own home is the preferred place of death. However, in the Netherlands more than 20% of deaths occur in the hospital [4–6]. In 2019, 32.846 patients died in Dutch hospitals [2]. A percentage of these patients suffered from conditions which may require palliative care (e.g. cancer, chronic life-threatening heart and lung conditions). In this article, we define palliative care as “the care for people with an incurable disease, aimed at improving quality of life by prevention and alleviation of suffering” [7]. However, patients without a condition relevant for palliative care can also die in hospital, for instance patients who die of complications of curative treatment or of an acute life-threatening condition.

As compared to palliative care delivered at home or in a hospice the quality of palliative care in the hospital is rated to be less, perceived by relatives [8]. Patient safety is an important element of quality of care, but patient safety in palliative care has received little attention so far [9]. Moreover, the majority of the limited available research only focused on the narrower group of patients receiving palliative/terminal care, and do not make comparisons with other patients at the end-of-life [9, 10], e.g. - as stated earlier - the patient without condition relevant for palliative care who dies in hospital. Hence, the aim of this study was to gain insight in patient safety for patients with a condition relevant for palliative care who died in hospital compared to other deceased hospital patients. More specifically, the following research questions were formulated:

1. What is the prevalence of (preventable) AEs and preventable deaths in patients with a condition relevant for palliative care and who died in the hospital? To what extent do these outcomes differ from deceased hospital patients who were admitted without a condition relevant for palliative care?
2. What are the nature, causes and prevention strategies of (preventable) AEs in patients who died in a hospital with a condition relevant for palliative care compared to deceased hospital patients who were admitted without a condition relevant for palliative care?

## Methods

### Study design

#### Design and sample

Data collection of this study was part of the Dutch Adverse Events Monitor, a longitudinal retrospective record review study among patients who died in Dutch hospitals [3]. A stratified sample was drawn of twenty hospitals, including university (*n* = 4), tertiary teaching (*n* = 6) and general hospitals (*n* = 10), stratified for region. University hospitals were oversampled to allow comparison between hospital types, the results were weighted to correct for this.

Per hospital, approximately 150 electronic patient records of patients who died in the hospital in 2019 were randomly selected from the hospital information system. Records from patients admitted to the psychiatry or obstetrics department and of children younger than one year were excluded. In total 2,998 patient records were included. Comparison between characteristics of the sample and all patients deceased in Dutch hospitals showed that the sample was representative (regarding age, sex, length of admission) for the total population of deceased patients in Dutch hospitals.

The full information on the design and sample of the Dutch Adverse Events Monitor (e.g. power calculation, record selection and check for representativeness) can be found elsewhere [2, 11].

#### Ethics

The Medical Ethical Committee of the VU University Medical Center (IRB00002991) declared that the Medical Research Involving Human Subjects Act did not apply (reference no.2020.052). The requirement of individual informed consent was exempted, as this study was conducted within the conditions of the Dutch Healthcare Quality, Complaints and Disputes Act [12].

### Record review

#### Process

Retrospective patient record review is a thorough and internationally widely used method to measure AE rates [1]. Patient records were reviewed from the electronic health system in a two-stage review process. In stage 1 nurses collected background characteristics such as sex, age and Charlson comorbidity index (weighted score representing comorbidity severity) [13]. Reviewing nurses furthermore screened the patient records using a trigger list (appendix Table 1), which is based on the Harvard Medical Practice Study [14]. Records with at least one present trigger were eligible for stage 2 review. In stage 2 physicians systematically reviewed the patient records for AEs.

The extensively trained reviewer group consisted of 17 nurses and eight physicians (medical specialties: internal medicine, neurology and surgery). The training consisted of one full day of training in which the trainer focused on: the consistent use of the right definition and rating for adverse events and preventability, and the overall approach of the record review. The trainer was a medical specialist who was involved in the Dutch Adverse Events Monitor since the first study in 2004. All reviewers had access to handbooks with comprehensive information and clinical examples during their record review. In addition to the training and handbooks, multiple intervision meetings and peer coaching sessions were organized throughout the data collection. Moreover, reviewers could always request a double-check/discussion meeting with external experts when in doubt regarding a record review. All reviewers had to have extensive clinical experience (minimum of 5 years for nurse reviewers and 10 years for medical specialist reviewers). Reviewers did not review patient records from the hospitals they worked at during the study or had worked at in the past.

#### Interrater reliability (IRR)

The IRR was calculated as positive and negative agreement, for both review stages a random sample of approximately 10% of records was reviewed double blind. The IRR in both stages was deemed sufficient. Positive agreement for finding triggers in the first stage was 97.4%, negative agreement was 75.0%. Positive agreement for determining an AE was 63.0%, negative agreement was 75.4%.

### Outcomes and measurements

#### Main outcomes

The three main outcomes to answer our first research question were: AE, potentially preventable AE and potentially preventable deaths. An AE was determined based on three criteria: unintended injury; health consequence; causality. An AE was considered potentially preventable when the provided care fell below the current level of performance that can be expected of the healthcare professional and/or hospital. When a potentially preventable AE had a contribution to the patient’s death it was considered a potentially preventable death. The main outcomes with definitions and measurements can be found in Table 1.

All included patients died in hospital, however, not all these patients suffered an AE, and not every AE contributed to the patient’s death. Patients with a condition relevant for palliative care could still experience a preventable death, when it was the preventable AE that led to their death instead of their condition. In other words: their death was sooner than could be expected based on their prognosis, and caused by healthcare management instead of their condition.

**Table 1:** Main outcomes, definitions and measurements

Outcome and definition	Measurement
**Adverse event:** an unintended injury that results in prolonged hospital stay, disability or death, and is caused by healthcare management rather than disease.	1. Unintended injury • Measurement: yes; no 2. Health consequence; the unintended injury resulted in prolonged hospital stay, disability or death • Measurement: 1 = no health consequence 2 = minimal health consequence and/or recovery within a month 3 = moderate health consequence, recovery in 1 to 6 months 4 = moderate health consequence, recovery in 6 to 12 months 5 = chronic health consequence, disability 1–50% 6 = chronic health consequence, disability > 50% 7 = death 8 = cannot be assessed 3. Causality; it is caused by healthcare management, and not the disease • Measurement: 1 = no indication of causality by healthcare professional and/or organization 2 = minor to moderate indication of causality 3 = no probable causality, less than 50–50 chance, but close call 4 = more probable causality, more than 50–50 chance, but close call 5 = strong indication of causality 6 = certain indication of causality Adverse event is scored when: (unintended injury = yes) + (health consequence score > 1) + (causality score ≥ 4)
**Potentially preventable adverse event:** an adverse event caused by providing care below the current level of performance that can be expected of the healthcare professional and/or healthcare system.	• Preventability measurement: 1 = no indication of preventability by healthcare professional and/or organization 2 = minor to moderate indication of preventability 3 = probable preventability, less than 50–50 chance, but close call 4 = more probable preventability, more than 50–50 chance, but close call 5 = strong indication of preventability 6 = certain indication of preventability Non preventable adverse event: (adverse event) + (preventability score 1) Somewhat preventable adverse event: (adverse event) + (preventability score 2–3) Potentially preventable adverse event: (adverse event) + (preventability score 4–6)
**Potentially preventable death:** a preventable adverse event had a contribution to the patient’s death.	• Contribution measurement: 1 = death is not related to adverse event 2 = adverse event had a moderate contribution to death 3 = adverse event had a substantial contribution to death 4 = death was (in its entirety) caused by adverse event Potentially preventable death: (potentially preventable adverse event) + (contribution score 3–4)

#### Secondary outcomes

When an AE was scored, the reviewers further reviewed its nature and causes. Moreover, for the potentially preventable AEs prevention strategies were determined, both categorically and through open text. These outcomes answered our second research question, the definitions and measurements can be found in appendix Table 2.

#### Condition relevant for palliative care

To identify the subgroups we used the selection method of Etkind (2017) based on codes from the international classification of Disease-10 (ICD-10) [15]. Patients with one or more relevant ICD-codes were classified as ‘with a condition relevant for palliative care’, all other patients were classified as ‘without a condition relevant for palliative care’. Both the main diagnosis of the index admission and co-morbidity diagnoses were taken into account. Appendix Table 3 shows the ICD-10 codes relevant for palliative care. Patients classified into the ‘condition relevant for palliative care’ group, did not necessarily have to be in the terminal phase of their palliative condition, nor did they have to be admitted to receive palliative or terminal care.

The ICD-10 codes were not available in patient records, but retrieved from a national database. Dutch Hospital Data (DHD) is a Dutch non-profit organization specialized in national hospital data [16]. DHD is processing, managing, analyzing and benchmarking the data for hospitals. DHD has multiple databases, i.e. the National Basic Register Hospital care. This database contains – among other data – ICD-10 codes of all Dutch hospitals. Registration in this national database is obliged for all hospitals. DHD states that the database is a reliable source for scientific research and analyses. The ICD-10 data is collected and coded by medical coders of the hospitals. They code the ICD-10 codes based on: the complete EHR, discharge letters and lab results. Out of the 20 included hospitals, 18 hospitals agreed to data retrieval from DHD. For the two hospitals that did not, the patients have missing ICD-10 codes (*n* = 380, 12.7%) and could not be classified into a subgroup. The analyses of this missing subgroup is added to the appendix.

### Data analysis

Descriptive statistics were calculated for the total sample of deceased patients, and the subgroups of patients with and without a condition relevant for palliative care. Descriptive analyses weighed for hospital type were performed to calculate all outcomes. T-tests were performed to test for differences between subgroups on main outcomes. StataSE version 14.1 was used.

## Results

### Background characteristics

Of the 2,998 included patient records 2,370 (79.1%) had a condition relevant for palliative care and 248 (8.3%) did not have a condition relevant for palliative care. Table 2 shows the background characteristics of the total sample and subgroups. The group of patients with a condition relevant for palliative care included less females than the group without a condition relevant for palliative care (45.2% vs. 51.6%), and had more severe comorbidity compared to the patients without a condition relevant for palliative care (Charlson score ≥ 5 85.6% vs. 72.2%).

**Table 2:** Background characteristics

	Total	Patients with a condition relevant for palliative care	Patients without a condition relevant for palliative care	Missing condition
Number of patients, n(row %)	2,998	2,370 (79.1%)	248 (8.3%)	380 (12.7%)
Hospital type, n(col %)				
Academic	610 (20.4%)	281 (11.7%)	27 (10.9%)	302 (79.5%)
Tertiary teaching	894 (29.8%)	764 (32.2%)	90 (36.3%)	40 (10.5%)
General	1,494 (49.8%)	1,325 (55.9%)	131 (52.8%)	38 (10.0%)
Admission, n(col %)				
Acute	2,657 (88.6%)	2,118 (89.4%)	231 (93.2%)	308 (81.1%)
Elective	120 (4.0%)	89 (3.8%)	6 (2.4%)	25 (6.6%)
Transfer	221 (7.37%)	163 (6.9%)	11 (4.4%)	47 (12.4%)
Sex, female n(col %)	1,357 (45.3%)	1,070 (45.2%)	128 (51.6%)	159 (41.8%)
Age, median [IQR]	78 [69–85]	78 [69–85]	80 [70.5–88]	73 [63–82.5]
Length of stay, median [IQR]	4 [2–10]	4 [2–9]	3 [1–7]	5 [1–14]
Charlson, n(col %)				
Score 0	24 (0.8%)	13 (0.5%)	5 (2.0%)	6 (1.6%)
Score 1–2	103 (3.4%)	60 (2.5%)	13 (5.2%)	30 (7.9%)
Score 3–4	396 (13.2%	269 (11.4%)	51 (20.6%)	76 (20.0%)
Score ≥ 5	2,475 (82.6%)	2,028 (85.6%)	179 (72.2%)	268 (70.5%)
Number of high risk medications, median [IQR]	6 [4–8]	6 [4–8]	6 [3–8]	6 [3–8]
Underwent surgery, n(col %)	398 (13.3%)	287 (12.1%)	42 (16.9%)	69 (18.2%)

* The two hospitals that could not deliver ICD data were academic hospitals, which is why the proportion of academic admissions is higher in the missing condition subgroup. Did this not affect outcomes, which are weighted for hospital type

### Adverse events

There was no statistically significant difference in AE prevalence between the patients with and without condition relevant for palliative care (t= -1.45 (design df = 3,069), *p* = 0.148). However, the crude AE prevalence was 3.3% higher in patients with a condition relevant for palliative care. In these patients we found 383 AEs in 352 patients (one patient can suffer multiple AEs during admission), the prevalence is 15.3% (95% CI 13.8%-16.9%). In the patients without a condition relevant for palliative care we found 36 AEs in 32 patients, the prevalence is 12.0% (95% CI 8.4%-16.8%).

There was no difference in potentially preventable AE prevalence between the patients with and without condition relevant for palliative care (t = 0.03 (design df = 3,069), *p* = 0.975). In the patients with a condition relevant for palliative care we found 103 potentially preventable AEs in 99 patients, the prevalence is 4.3% (95% CI 3.5%-5.3%). In the patients without a condition relevant for palliative care we found 16 potentially preventable AEs in 14 patients, the prevalence is 4.4% (95% CI 2.5%-7.4%).

We found no difference in potentially preventable death prevalence between patients with and without condition relevant for palliative care (t= -0.08 (design df = 3,069), *p* = 0.938). In the patients with a condition relevant for palliative care we found 73 patients with a potentially preventable death, the prevalence is 3.2% (95% CI 2.5%-4.1%). In the patients without a condition relevant for palliative care there were 11 patients with a potentially preventable death, the prevalence is 3.2% (95% CI 1.7%-5.7%).

**Fig. 1:**
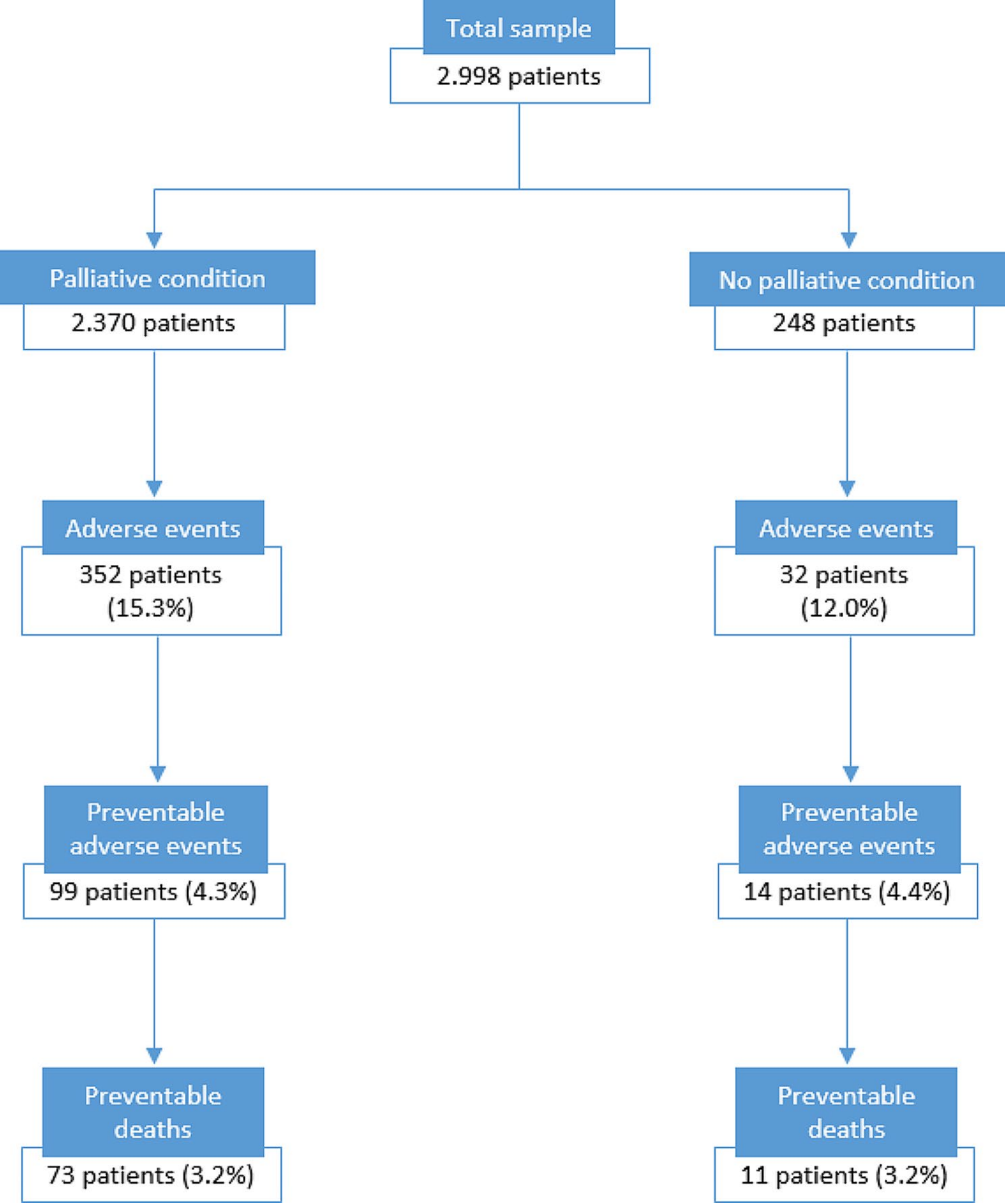
Flowchart of patients with adverse events in the subgroups, weighted percentages *Palliative condition = patients with a condition relevant for palliative care *No palliative condition = patients without a condition relevant for palliative care

Table 3 shows clinical examples of AEs in the two subgroups from our data. Figure 1 shows a flowchart of AEs. Appendix Table 4 shows the AEs, missing condition group included. We did not find different rates in the missing subgroup.

**Table 3:** Examples of adverse events

	Patients with a condition relevant for palliative care	Patients without a condition relevant for palliative care
Adverse event	Male patient of 78 years old, suffering advanced metastatic prostate cancer. Patient was admitted for, and died from complications of chemotherapy: leukopenia sepsis and disseminated intravascular coagulation.	Female patient of 63 years old, admitted for facial, skull and brain injury due to physical abuse. Patient had a central line placed in groin, which got infected. The blood culture showed a bacteraemia, for which the patient received antibiotics.
Preventable adverse event	Male patient of 71 years old, suffering from metastatic urothelial carcinoma. Patient was admitted with a pathological spine fracture. Pre-surgery the patient had low oxygen saturation, and a chest x-ray revealed pulmonary metastasis. Despite these contra-indications spondylodesis was performed, shortly after which the patient suffered respiratory problems and ICU admission followed. Patient further deteriorated and a palliative policy was decided upon in consultation with the relatives, after which the patient died.	Female patient of 57 years, initially got admitted due to drain dysfunction with a pseudomonas infection. Patient underwent nephrectomy through laparoscopy. Two days later she suffered a colon perforation for which more surgical procedures were performed and abdominal sepsis and need for a stoma followed. The course of the patient’s total admission was long and intense: more than four months with 10 + surgical procedures. She eventually died, cause of death was unclear.
Preventable death	Female patient of 81 years old, was admitted for a Cerebro Vasculair Accident (CVA). Patient underwent surgical thrombectomy, which failed. After stabilisation the patient received thrombolysis despite contra-indications, causing multiple bleedings and eventually death.	Female patient of 81 years old, suffering peripheral vascular disease got admitted for the symptoms. Patient underwent PTA (Percutaneous Transluminal Angioplasty) after which a heavy bleeding followed. The bleeding was not treated or stopped (for which the reason was not described in the patients record), causing death.

### Nature

Table 4 shows the nature of AEs for both subgroups. Of the 383 AEs in patients with a condition relevant for palliative care, most were related to medication (*n* = 133, 33.1%). These AEs were related to different medication categories, but most frequent were anticoagulation (*n* = 50) and chemotherapy (*n* = 39).

Of the 36 AEs in the patients without a condition relevant for palliative care, most were related to surgery (*n* = 17, 50.8%). The AEs were related to different surgical procedures, but most frequent was implants for hip fractures (*n* = 7).

**Table 4:** Nature of adverse events

	Patients with a condition relevant for palliative care	Patients without a condition relevant for palliative care
Diagnostic	25 (6.1%)	4 (10.5%)
Surgery	69 (18.2%)	17 (50.8%)
Non-surgical medical procedure	48 (14.6%)	2 (4.3%)
Medication	133 (33.1%)	6 (14.8%)
Other clinical management	97 (24.8%)	4 (11.1%)
Discharge	1 (0.2%)	0
Other	10 (3.1%)	3 (8.6%)
	Total: 383 adverse events	Total: 36 adverse events

*Percentages are weighted for hospital type

### Causes

**Fig. 2:**
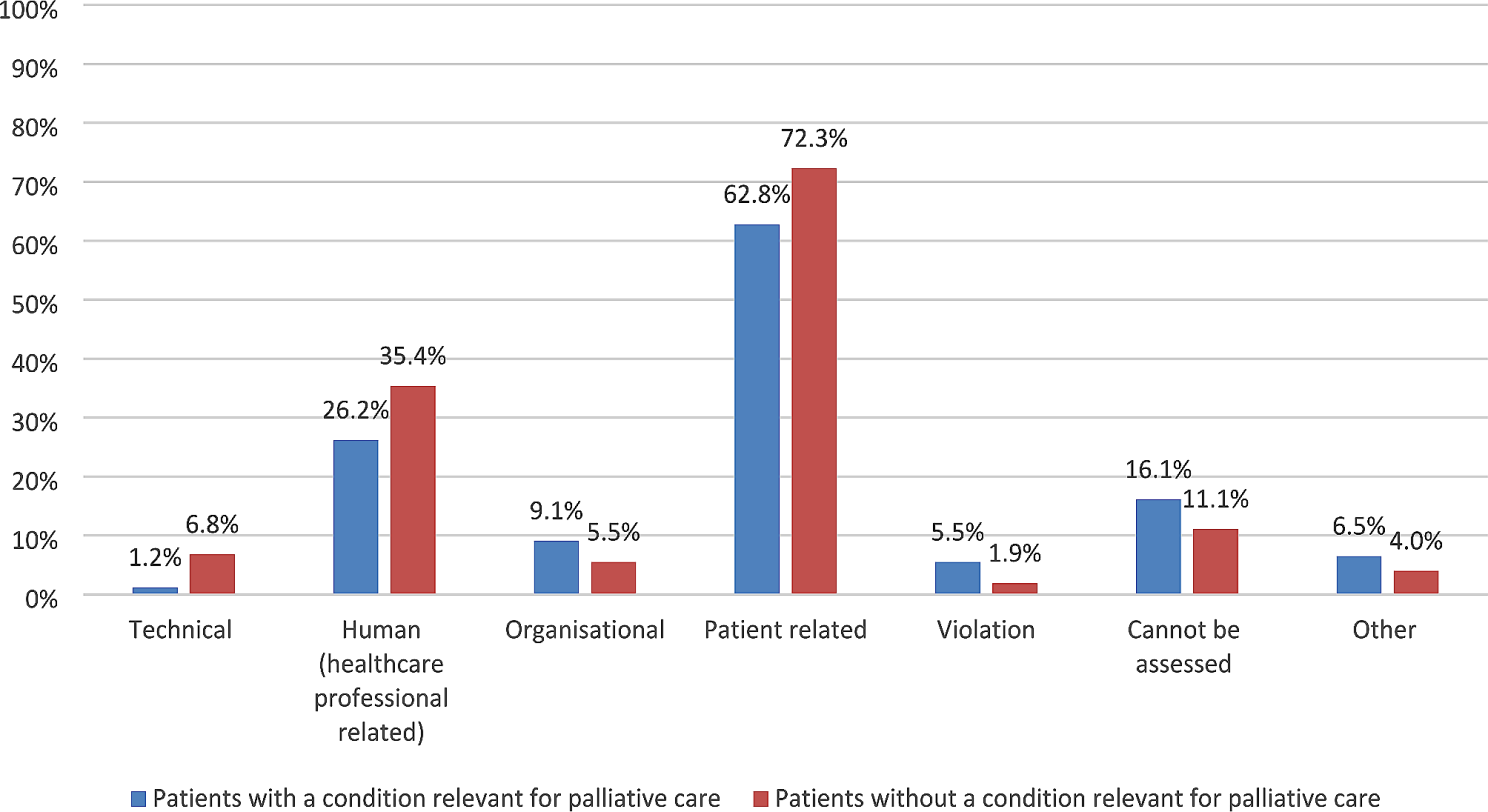
Causes of adverse events *Percentages do not add up to 100% as one adverse event could have multiple causes *Percentages are weighted for hospital type

Figure 2 shows the causes of AEs for both subgroups, one AE could have multiple causes. For both subgroups the majority of AEs had a patient related cause: 62.8% of AEs in patient with a condition relevant for palliative care, and 72.3% of AEs in patients without a condition relevant for palliative care. Of the patient related AEs the most frequent sub-cause in both subgroups was comorbidity (94.8% and 97.0% respectively).

### Prevention strategies

Table 5 shows the frequencies of prevention strategies in both subgroups, including an example from the open text fields in the data. These prevention strategies were suggested by the medical reviewers. The most occurring prevention strategies did not differ much between patients with or without a condition relevant for palliative care. Of the 103 potentially preventable AEs in patients with a condition relevant for palliative care, the top three of categorical prevention strategies were: reflection and evaluation (*n* = 78), quality assurance (*n* = 48) and procedures (*n* = 25). Of the 16 potentially preventable AEs in patient without a condition relevant for palliative care, the top three was: reflection and evaluation (*n* = 10), quality assurance (*n* = 6) and training (*n* = 6).

**Table 5:** Prevention strategies of preventable adverse events

Prevention strategy with open text examples	Patients with a condition relevant for palliative care	Patients without a condition relevant for palliative care
Procedures	25	4
Information and communication	8	1
Training	16	6
Motivation	12	2
Quality assurance	48	6
Scaling up	6	4
Reflection and evaluation	78	10
Other	3	0
	Total: 103 preventable adverse events	Total: 16 preventable adverse events

*The following categories were not selected (*n* = 0) as prevention strategies: technology, financial, staff

*For one preventable adverse event, multiple prevention strategies could be selected

The open texts of these categorical prevention strategies showed more in-depth information on how the preventable AEs may have been prevented. When *reflection and evaluation* and/or *quality assurance* was selected as prevention strategy, suggestions in the open text fields included: to conduct complication- and necrology discussion meetings, and to reflect upon and learn from complex cases. This did not differ between subgroups. Many of the AEs were regarding deterioration in a patient that was not or too late recognized.

*Procedures* was in the top three for patients with a condition relevant for palliative care, open texts mostly described whether performed medical procedures (e.g. medication treatment or surgeries) should have been performed in this subgroup, and to have more attention for the frail pre-existing status. *Training* was in the top three for patients without a condition relevant for palliative care and the open text fields mostly suggested technical training in performing (surgical) procedures.

## Discussion

This study identified the prevalence of AEs and its preventability, nature, causes and prevention strategies in deceased patients with and without a condition relevant for palliative care. Our study identified no statistically significant difference in AE prevalence, potentially preventable AE prevalence and potentially preventable death prevalence between patients with a condition relevant for palliative care compared with other deceased hospital patients. This indicates that safety threats for patients who die in hospital are equally prevalent for patients with as patients without condition relevant for palliative care. Though comparable literature on patient safety in hospital patients at the end of life is lacking, this was contrary to our expectations. We formerly expected patients with condition relevant for palliative care to be at higher risk of suffering AEs, as previous research.

has shown that palliative patients are more frail on a physical and psychosocial level, reducing their resilience [10, 17–19]. However, it could be that patients who died in the hospital without a condition relevant for palliative care undergo more high risk (curative treatments) compared to patients with condition relevant for palliative care, exposing them to potential AE risk.

Although we did not find differences in prevalence, we did find that the nature of AEs differed between the two subgroups (medication versus surgery-related). This indicates that tailored safety measures are needed. Of the AEs in patients with a condition relevant for palliative care 33.1% related to medication, whereas this was only 14.8% in patients without a condition relevant for palliative care. The median number of high risk medications did not differ between the subgroups (median = 6 in both groups). This indicates that while they receive the same number of high risk medications, patients with conditions relevant for palliative care are at higher risk to suffer medication AEs. This is in line with previous research showing that patients with a condition relevant for palliative care relatively often receive drug treatment to alleviate pain and other symptoms, with a high risk of medication errors [10, 20]. Moreover, patient safety research in palliative care settings shows high rates of medication errors [10, 17, 21, 22]. Although anticoagulation is known to be one of the medication categories with the highest risk of AEs, our results show that in particular patients with a condition relevant for palliative care can be at higher risk. This might be due to the premorbid risk factors caused by severe comorbidity. Research by Arevalo et al. showed that 72.9% of palliative patients receive antithrombotic agents until the last days of life, whereas this is only 27.1% in hospice. Hospice medication management might be more attentive and fitting for patient at their end-of-life [23]. The Dutch national safety-II movement ‘time-to-connect’ offers a range of best practice examples regarding anticoagulation in the hospital setting, though not specifically for this vulnerable group of patients with a condition relevant for palliative care hospitalized at the end-of-life [24]. Further research is needed with regard to which of these or other safety measures can help reducing anticoagulation harm specifically in this group, and to guide healthcare professionals in hospitals in balancing out the pros and cons of using anticoagulation in these patients. The AEs in patients without a condition relevant for palliative care were mostly related to surgery, namely in 50.8%, whereas this was only 18.2% in patients with a condition relevant for palliative care. This could partially be explained by the fact that more patients without a condition relevant for palliative care underwent surgery (16.9% vs. 12.1%), and were therefore exposed more to surgical risks. The surgical AEs in patients related to different surgical procedures, but most frequent to implants for hip fractures. This is in line with previous research showing a high risk of AEs in hip fracture patients [25]. Mainly patients with severe comorbidity suffer complications [26]. Though AEs in this frail group might be challenging to prevent, research shows that a high ASA-score can predict complications in hip fracture patients. Risk awareness is one important factor in preventing these complications [27].

In both subgroups the majority of AEs had a patient related cause. A patient related cause implies that patient related factors – e.g. comorbidity, patient preferences or communication skills – contributed to the origin of the AE. In this study we made an inventory of all the potential causes contributing to the AEs, also the non-preventable AEs. The most frequent sub-cause was comorbidity. From a patient safety perspective, it is not the goal – or potentially possible – to prevent all comorbidity and preventing AEs in frail patients with a high level of comorbidity might not always be possible. However, it is very important that comorbidity is taken into consideration in medical decision making, both by healthcare professionals and patients. An important condition is a comprehensive and real-time overview of comorbidities in EHRs. Research from the UK shows that the quality of clinical coding in the NHS was suboptimal, particularly with regards to the recording of comorbidities [28].

For the potentially preventable AEs in both subgroups the two most important prevention strategies as suggested by the medical reviewers were *reflection and evaluation* and *quality assurance.* Many of the preventable AEs where these strategies were selected related to not or delayed recognition of deterioration in patients. This indicates that rapid deterioration is not easily recognized by healthcare staff, independently of whether or not a patient has a condition relevant for palliative care. More learning based on reflection might prevent these AEs in the future.

### Limitations

Even though retrospective review is the most widely used method in AE monitoring, it can be affected by hindsight bias because the reviewers know the negative outcomes, i.e. death, at the moment of review. This could have resulted in overestimation of AE rates and/or degree of preventability. Moreover, the results are based on the information that was available in the EHRs, meaning that the reviewers did not always have access to all the potentially relevant information. Secondly, the subgroups were identified based on ICD-10 codes. This could be subject to bias, as some patients might have had certain diagnoses relevant for palliative care, that were not registered in the EHR. Therefore, the group with patients with a condition relevant for palliative care might be even larger. However, we do expect the potential impact of this bias to be relatively small, as the ICD-10 codes were retrieved from the National Basic Register Hospital care, which is deemed to be a reliable database. The ICD-10 codes from this database are deemed to be more complete than studies only using ICD-10 codes directly retrieved from (the problem list of) the EHR, as they are carefully coded based on multiple sources. A group of *n* = 380 could not be classified into the subgroups due to missing ICD-10 codes. These were missing because two hospitals did not approve data retrieval from DHD, due to privacy reasons. These were two university hospitals. Therefore, we analysed the main outcomes in the missing subgroup and did not find any potentially impactful differences compared to the outcomes of the other subgroups.

### Implications

Although we did not find any statistically significant differences on the main outcomes, we did find a crude higher AE risk in patients with a condition relevant for palliative care. In this broad group of patients with a condition relevant for palliative care, the palliative phase might not always be easily recognized [29]. Therefore, it could help healthcare professionals to be extra attentive to the premorbid frail state in all hospitalized patients admitted with a condition relevant for palliative care, even when death is not initially expected or impending when admitted.

Although many AEs we found in this study in both subgroups were deemed non-preventable, it can be a thin line between what is or is not preventable. Therefore, it is important to not only learn from potentially preventable AEs, but also from non-preventable AEs. This can be complemented with safety-II approaches, e.g. by having medical teams reflect on why and how processes go right in certain situations and wrong in others.

Lastly, many AEs occurred because deterioration was not recognized, or too late in patients who died during hospitalization. A recommendation is to train and further strengthen the situational awareness of healthcare professionals.

## Conclusion

This study found no statistically significant difference in AEs, potentially preventable AEs and potentially preventable death prevalence between patients with and without a condition relevant for palliative care. The nature of AEs did differ between subgroups. We found mainly medication related AEs in patients with condition relevant for palliative care, and surgical related AEs in patients without condition relevant for palliative care. This suggests that tailored safety measures are needed. Implications for practice and future research is e.g. medication management attentive and fitting to patients at the end of life, and to focus on learning from and reflecting on AEs.

### Electronic supplementary material

Below is the link to the electronic supplementary material.

Supplementary Material 1

## Data Availability

Data are available upon reasonable request after approval of the corresponding author.
