# Supplementary material for: Adverse events at the end of life of hospital patients with or without a condition relevant for palliative care: a nationwide retrospective record review study in the Netherlands
Source: BMC Palliat Care. 2024 Jun 10;23:145. doi: 10.1186/s12904-024-01461-z (PMC11163706; doi:10.1186/s12904-024-01461-z)
Supplement: Supplementary file 1 — Supplementary Material 1 [file 12904_2024_1461_MOESM1_ESM.docx]

# Supplementary content - Adverse events in hospitals among patients with or without a condition relevant for palliative care: a nationwide retrospective record review study

B. Schouten MSc, S.M. van Schoten PhD, F.M. Bijnsdorp PhD, H. Merten, PhD, P.W.B. Nanayakkara PhD, A.K.L. Reyners PhD, A.L. Francke PhD, C. Wagner PhD

# Contents:

Title page and contents Page 1

Appendix table 1. Trigger list Page 2

Appendix table 2. Secondary outcomes, definitions and Page 3/4

measurements

Appendix table 3. International Classification of Disease-10 Page 5

codes indicating conditions relevant for palliative care

Appendix Table 4. Adverse events, missing condition Page 5

group included

**Appendix table 1.** Trigger list

| No. | Trigger |
| --- | --- |
| 1 | Patient was admitted before (<12 months) index admission for reasons related to the index admission |
| 2 | Hospital acquired permanent or temporary injury during index admission |
| 3 | Adverse drug reaction |
| 4 | Unplanned transfer from a general ward to the intensive care unit (including cardio care unit) |
| 5 | Unplanned transfer to another hospital after unexpected deterioration of the patient |
| 6 | Unplanned (revision) surgery |
| 7 | Unplanned removal, injury or repair of an organ or tissue during surgery or invasive procedure |
| 8 | Hospital acquired infection or sepsis during index admission (excluding infections/sepsis acquired within the first 72 hours of admission) |
| 9 | Other complications, for example acute myocardial infarction, TIA/CVA, pulmonary embolism, etc. (Encompasses every unexpected complication that is not a natural cause if the disease of the patient or an expected outcome of the treatment) |
| 10 | Neurological deficit developed during index admission |
| 11 | Unexpected death of the patient (excluding patients admitted for palliative care) |
| 12 | Cardiac or respiratory arrest (with successful resuscitation) |
| 13 | Dissatisfaction with the provided care documented in the patient record and/or an indication of filed complaints (including documented complaints, conflicts between patient/family and hospital staff) |


**Appendix table 2.** Secondary outcomes, definitions and measurements

| Outcome and definition | Measurement |
| --- | --- |
| Nature: what was the nature of the adverse event, i.e. what clinical process was the adverse event mostly related to? | - Measurement:   1 = diagnostic (adverse event related to a misdiagnosis, missed or delayed diagnosis)  2 = surgery (adverse event related to a surgical procedure – during surgery or within 30 days post-surgery)  3 = non-surgical medical procedure (adverse event related to a non-surgical medical procedure, e.g. endoscopic procedures)  4 = medication (adverse event related to medication)  5 = other clinical management (adverse event related to other (nursing) clinical activities, including nursing- and paramedical care)  6 = discharge (adverse event related to discharge)  7 = other (adverse event did not fall within any of the other categories, e.g. accidents like burns and falls)  For each adverse event one main category was selected for nature, i.e. one adverse event related mostly to one clinical process. |
| Causes: what factors caused the adverse event? | - Measurement:   1 = Technical (errors relating to the design or construction of medical equipment, software, materials, labels or forms)  2 = human – healthcare professional related (errors relating to knowledge, rule-based behavior or skills of healthcare professionals)  3 = Organizational (errors relating to protocols, procedures, information transfer, culture or management priorities)  4 = patient related (errors relating to characteristics of the patient i.e. comorbidity, age, communicative skills, (demanding) personality or therapy compliance)  5 = violation (errors relating to negligence or carelessness)  6 = cannot be assessed  7 = other  For each adverse event all factors that caused it could be selected, i.e. one adverse event could have multiple causes. |
| Prevention strategies: in what areas or which ways is effort needed to prevent recurrence of this adverse event in the future? | - Measurement, categorical:   1 = Technology (redesign of equipment etc.)  2 = Procedures (completing or improving formal and informal procedures)  3 = Information and communication (completing or improving existing information sources, communication structures and filing)  4 = training (improving training programs for required skills)  5 = motivation (increasing the level of voluntary obedience to generally accepted rules by positive behavioral change)  6 = quality assurance (continuous monitoring of quality data based on pre-specified standards, and assessment of healthcare professionals performance by individuals in the same field)  7 = scaling up (handling issues at a higher organizational level, like the hospital management)  8 = reflection and evaluation (reflecting on and evaluating the current way of behaving regarding safety)  9 = financial (financial investment)  10 = staff (more staff)  11 = other   - Measurement, open text field:   Description of why the adverse event was preventable in that particular case, and which actions or measures could have prevented it from happening. This field was filled out for all potentially preventable adverse events.  For each preventable adverse event all measurements that could have prevented the AE were selected, i.e. one preventable adverse event could have multiple prevention strategies. |

**Appendix table 3**. International Classification of Disease-10 codes indicating conditions relevant for palliative care (Etkind, 2017)

| Group | Code | Conditions included |
| --- | --- | --- |
| Cancer | C00–C97 | All deaths from malignant neoplasms. |
| Organ failure | I00–I52 (excl. I12 & I13) J40–J47, J96 I12, I13, N17, N18, N28 K70–K77 | Heart disease and heart failure; Chronic lower respiratory disease, respiratory failure  Reno-vascular disease, renal failure;  Liver disease. |
| Dementia | F01, F03, G30, R54 | Dementia, vascular dementia, Alzheimer’s disease, senility. |
| Other | G10, G12.2, G20, G23.1, G35, G90.3 I60–I69 B20–B24 | Huntington’s disease, motor neurone disease, Parkinson’s disease, progressive supranuclear palsy, multiple sclerosis, multi system atrophy, hemorrhagic, ischemic and unspecified stroke;  HIV |

**Appendix table 4.** Adverse events, missing condition group included

|  | Total | Patients with a condition relevant for palliative care | Patients without a condition relevant for palliative care | Missing condition |
| --- | --- | --- | --- | --- |
| Number of patients, n(row %) | 2,998 | 2.370 (79.1%) | 248 (8.3%) | 380 (12.7%) |
| Patients with an adverse event, n (weighted %) | 435 (14.6%) | 352 (15.3%) | 32 (12.0%) | 51 (12.0%) |
| Patients with a preventable adverse event, n (weighted %) | 127 (4.2%) | 99 (4.3%) | 14 (4.4%) | 14 (3.2%) |
| Patients with a preventable death, n (weighted %) | 95 (3.1%) | 73 (3.2%) | 11 (3.1%) | 11 (2.5%) |
